# Aspergillosis with pulmonary echinococcosis

**DOI:** 10.1186/1742-6413-3-7

**Published:** 2006-03-30

**Authors:** Nalini Gupta, Julie Arora, Raje Nijhawan, Ritesh Aggarwal, Anupam Lal

**Affiliations:** 1 Assistant Professor, Department of Cytopathology, Postgraduate Institute of Medical Education and Research, Chandigarh, INDIA; 2 Senior Resident, Department of Radiodiagnosis, Postgraduate Institute of Medical Education and Research, Chandigarh, INDIA; 3 Additional Professor, Department of Cytopathology, Postgraduate Institute of Medical Education and Research, Chandigarh, INDIA; 4 Assistant Professor, Department of Pulmonary Medicine Postgraduate Institute of Medical Education and Research, Chandigarh, INDIA; 5 Associate Professor, Department of Radiodiagnosis, Postgraduate Institute of Medical Education and Research, Chandigarh, INDIA

Pulmonary echinococcal hydatid cysts have been reported coexistent with cryptococcosis and other saprophytic mycosis on histopathological examination of the affected lung tissues [1-3]. Similar findings have not been reported in Fine needle aspiration (FNA) samples. The authors describe a similar case diagnosed on FNA material.

**Figure 1:**
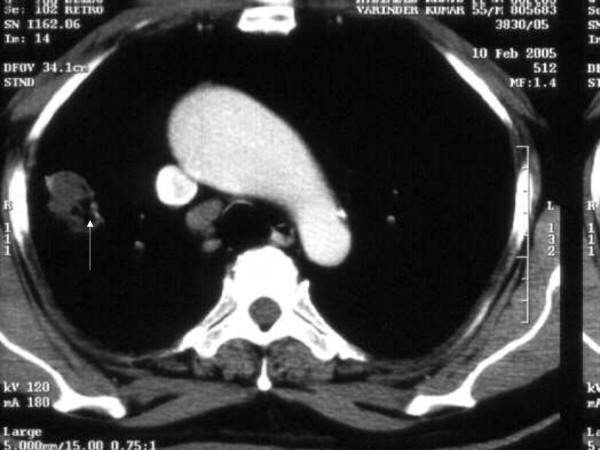
CT scan showing a solitary pulmonary nodule (arrow) with focal areas of cavitation and spiculated margins in right lung.

**Figure 2:**
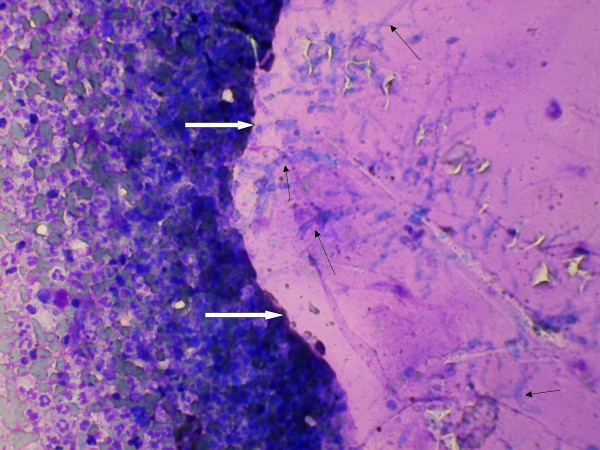
Microphotograph showing septate branching fungal hyphae (black arrows) embedded on the laminated ectocyst of echinococcus (Thick white arrows) (MGG × 512).

**Figure 3:**
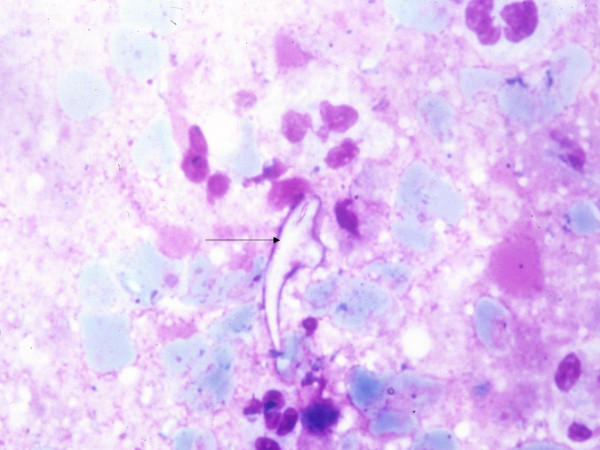
Microphotograph showing a hooklet of hydatid (arrow) (MGG × 1375).

A 53-year-old male, known diabetic for the last five years, presented with history of cough and fever with occasional haemoptysis for the last three months. A chest X-ray showed an oval homogeneous density in the upper lobe of right lung. Results of laboratory investigations such as haemogram, routine urine and stool examinations and sputum examination did not reveal any abnormality. He was non-reactive for Human Immunodeficiency Virus (HIV). Computed Tomography (CT) scan showed a solitary pulmonary nodule measuring about 2.5 × 2 × 1 cm with focal areas of cavitation and spiculated margins in right lung (Figure 1). CT guided fine needle aspiration (FNA) was performed from this solitary pulmonary nodule. The material aspirated was blood mixed pus like material. The smears were cellular and composed of sheets of polymorphs and histiocytes. There were scattered fragments of acellular homogenous laminated membranous structures representing laminated ectocyst of echinococcus. Also fungal hyphae, which were of uniform width, septate and branching morphologically consistent with Aspergillosis were identified. These hyphae were seen embedded on the laminated ectocyst (Figure 2). The smears also showed many scattered hooklets of hydatid (Figure 3) along with few multinucleated giant cells. Therefore, he was diagnosed to have concurrent Aspergillosis with pulmonary Echinococcosis by CT guided FNA from pulmonary nodule. The fungal culture of the aspirated material was not done in the present case.

Coexistence of fungi with pulmonary echinococcal hydatid cysts has occasionally been reported in the English literature, but similar findings have not been reported in FNA samples. The fungus in these cases can invade the outer aspect of the laminated membrane and can grow in the cavity of the pericyst. Coexistence of fungi with pulmonary hydatid cyst is seen more commonly in immuno-compromised patients. In the present case, the patient was a known diabetic and was taking oral hypoglycemic drugs for the last five years. This case demonstrates (on FNA material) invasion of the laminated membrane (ectocyst) of hydatid by a fungus seen as hyphae branching dichotomously at acute angles morphologically consistent with Aspergillosis. The same has not been reported in the English literature previously. The aim of this communication is also to highlight the atypical clinical presentation, radiological findings and importance of CT guided FNA to arrive at an accurate diagnosis. The last point merits underscoring from the viewpoint of the immuno-compromised patient, in whom, hydatid disease may coexist with other pathogens, such as fungi and these pathogens need to be accurately identified in radiologically guided FNA. It is also important to bear in mind that rarely, hydatid fluid can incite an anaphylactic reaction during the procedure. Adequate emergency measures should be available at hand to circumvent any untoward effects resulting from such a reaction.
